# What do antenatal care providers understand and do about oral health care during pregnancy: a cross-sectional survey in New South Wales, Australia

**DOI:** 10.1186/s12884-016-1163-x

**Published:** 2016-12-01

**Authors:** Ajesh George, Hannah G Dahlen, Jennifer Reath, Shilpi Ajwani, Sameer Bhole, Andrew Korda, Harrison Ng Chok, Charmaine Miranda, Amy Villarosa, Maree Johnson

**Affiliations:** 1 Collaboration for Oral Health Outcomes, Research Translation and Evaluation (COHORTE) Research Group, Western Sydney University, South Western Sydney Local Health District, Ingham Institute Applied Medical Research,University of Sydney, Locked Bag 7103, Liverpool, 1871 Australia; 2 School of Nursing & Midwifery, Western Sydney University, Ingham Institute Applied Medical Research, Parramatta, 2150 Australia; 3 Peter Brennan Chair of General Practice, School of Medicine, Western Sydney University, Parramatta, 2150 Australia; 4 Sydney Local Health District Oral Health Services, Sydney Dental Hospital, University of Sydney, Sydney, 2010 Australia; 5 Sydney Local Health District Oral Health Services, Sydney Dental Hospital, University of Sydney, Sydney, 2010 Australia; 6 Obstetrics and Gynaecology, School of Medicine, Western Sydney University, Parramatta, 2150 Australia; 7 Centre for Nursing Research and Practice Development, Western Sydney University, Nepean Blue Mountains Local Health District, Penrith, 2751 Australia; 8 College of Professional Psychology, Crows Nest, 2065 Australia; 9 COHORTE Research Group, Western Sydney University, South Western Sydney Local Health District, Ingham Institute Applied Medical Research, Liverpool, 1871 Australia; 10 Faculty of Health Sciences, Australian Catholic University, Ingham Institute Applied Medical Research, Sydney, 2060 Australia

**Keywords:** Perinatal, Antenatal, Oral health, Dentists, Pregnant women, Antenatal care

## Abstract

**Background:**

There is mounting evidence to support the lack of awareness among pregnant women about health consequences and long term risks associated with poor oral hygiene during pregnancy. A recognised and important point of influence is their interaction with health professionals, particularly when receiving Antenatal Care. However, there is limited evidence about the perceptions of ANC providers in Australia toward the provision of perinatal oral healthcare. This study was undertaken to explore the knowledge, attitudes and practices of Antenatal Care (ANC) providers in New South Wales (NSW), Australia providing perinatal oral healthcare and to identify barriers to and predictors of their practices in this area.

**Methods:**

A cross sectional survey was undertaken of ANC providers (general practitioners, obstetricians/gynaecologists and midwives) practising in NSW, Australia. Participants were recruited through their professional organisations via email, postal mail, and networking at conferences. The survey addressed the domains of knowledge, attitude, barriers and practices towards oral healthcare, along with demographics. Data was entered into SPSS software and analysed using descriptive and inferential statistics.

**Results:**

A total of 393 surveys (17.6% response rate) were completed comprising 124 general practitioners, 74 obstetricians/gynaecologists and 195 midwives. The results showed limited knowledge among ANC providers regarding the impact of poor maternal oral health on pregnancy/infant outcomes. Most (99%) participants agreed that maternal oral health was important yet few were discussing the importance of oral health or advising women to visit a dentist (16.4–21.5%). Further, less than a third felt they had the skills to provide oral health advice during pregnancy. ANC providers who were more knowledgeable about maternal oral health, had training and information in this area and greater experience, were more likely to engage in practices addressing the oral health of pregnant women.

**Conclusion:**

The findings suggest that ANC providers in NSW are not focussing on oral health with pregnant women. ANC providers seem willing to discuss oral health if they have appropriate education/training and information in this area. Further research at a national level is required to confirm whether these findings are similar in all Australian states.

## Background

Poor maternal oral health can negatively impact on both maternal and foetal health and wellbeing. Hormonal variations put pregnant women at a higher risk of developing gum disease [1, 2] which has been associated with various adverse pregnancy outcomes, including low birth weight and premature birth [3–5]. Mothers with untreated dental decay are also more likely to pass on the bacteria that causes tooth decay to their child after birth, through the direct transmission of their infected saliva, especially if they are sharing the same spoon while feeding the child [6].

However, many expectant mothers lack awareness of the impact of their own oral health on their baby’s health. Even when they are aware that they have dental problems, only a third of pregnant women in Australia consult a dentist during pregnancy [7, 8]. The use of dental services by pregnant women is consistently low internationally, including the United States of America (USA) (23–49%), United Kingdom (UK) (33–64%) and Greece (27%) [9–11]. This low uptake of dental services has primarily been linked to various barriers pregnant women face, such as cost, lack of public awareness of the importance of dental health, myths about the effects of pregnancy on dental health, and concerns for fetal safety during dental treatment services [12–15].

To address this, it is now recommended in Australia and internationally that all Antenatal Care (ANC) providers provide oral health education, assessments and referrals during pregnancy [16]. The potential for ANC practitioners to provide preventative oral health services has been recognised in many countries [17–20]. In the UK, ANC practitioners strongly recommend all pregnant women to visit their dentist and seek free treatment under the National Health Service (NHS) [21]. In the USA, oral health guidelines focusing on education, assessment and referrals in their antenatal practices have been incorporated into various maternity programs [17, 18]. Similar initiatives have been undertaken in Australia, including developing evidence based oral health promotion material (NSW Health, 2010), providing oral health education to midwives [22] and incorporating oral health guidelines into current midwifery practice through the Midwifery Initiated Oral Health (MIOH) program [23].

However, in order to raise awareness among pregnant women and increase their use of dental services, consensus must be reached among all ANC providers in this area. A recent review revealed that many general practitioners have the misconception that dental procedures are unsafe during pregnancy [24]. While obstetricians/gynaecologists are well informed about maternal oral health and are supportive of dental procedures, they lack the time and training to focus on oral health care during antenatal care. This lack of training and consensus can be a significant deterrent for pregnant women seeking dental care.

In Australia there is limited research concerning the perceptions of ANC providers about oral health care during pregnancy [25]. The only published study, a qualitative study involving midwives, highlighted there was a knowledge gap in this area [25]. Further research on the current perceptions and practices of ANC providers is needed to better understand existing perceptions and practices and identify ways to address identified gaps in ANC providers’ understanding. This study was part of a larger study also exploring perceptions of dentists in Australia presented elsewhere (George et al., in progress).

### Aim

The aim of this study was to explore the knowledge, attitudes and practices of Antenatal Care (ANC) providers in New South Wales (NSW), Australia providing perinatal oral healthcare and to identify barriers to and predictors of their practices in this area. The research questions include the following:

- How knowledgeable are ANC providers about oral health care during pregnancy?
- What are the attitudes of ANC providers towards oral health care during pregnancy?
- Do ANC providers engage in perinatal oral health practices with pregnant women?
- What are the barriers to ANC providers discussing oral health care during pregnancy?
- What factors predict whether ANC providers discuss oral health and provide referrals to pregnant women?

## Methods

### Sample and setting

A cross-sectional survey of a sample of ANC providers including general practitioners (GPs), obstetricians and gynaecologists (O&Gs), and midwives practising in New South Wales (NSW) was conducted from October 2013 to June 2014.

### Survey design

An online survey was created using Qualtrics [26]. Review of the literature, policy statements and other international guidelines and expert opinion regarding dental care during pregnancy was involved in the development of the survey content. Most of the survey items were derived from existing questionnaires exploring perceptions of ANC providers regarding perinatal oral health [19]. Questions were presented in four domains which included knowledge about perinatal oral health, attitude and practices related to oral health promotion, and barriers to providing care. Demographic data including personal and practice information was also collected. The survey design consisted of multiple-choice questions (true/false/not sure) in the knowledge domain and likert scales (1 strongly agree – 5 strongly disagree) to assess the attitudes, practices and barriers of the ANC providers. The survey was reviewed by experts to establish content validity and then tested for online access and readabilty by a group of ANC providers practising in a large metropolitan health district. Survey content, clarity and length were modified and improved in response to the review.

### Recruitment and data collection

ANC providers were recruited through their professional organisations via email, postal mail, networking and at conferences. The professional organisations included the Australian Medical Association (AMA), the Royal Australian College of General Practitioners (RACGP), the Australian College of Midwives (ACM) and the Royal Australia and New Zealand College of Gynaecologists (RANZCOG).

Emails with an online link to the survey were initially sent via the professional bodies to 826 GPs, 1436 midwives and 305 O&Gs inviting them to participate in the survey. The email also included an information sheet with details of the study and the option of completing the survey through reply paid postal mail. A reminder email with the information sheet and link was sent approximately one month later via the professional bodies. Midwives and O&Gs were also approached during local and national conferences.

### Data analysis

Data from the survey were analysed using SPSS Version 22 [27]. To explore the knowledgability, attitudes, practices and barriers of ANC providers regarding oral health, descriptive statistical analyses were used. Correct responses from the questions in the Knowledge domain were aggregated into a score out of 25. Continuous data were tested for normality using the Shapiro-Wilk test. Any nonparametric continuous variables were recoded into ordinal variables for ease of analysis. To explore factors that predict whether ANC providers discuss oral health and provide referrals to pregnant women, contingency tables with Pearson’s chi-squared tests were first conducted to examine relationships between variables of interest and other factors. For the purpose of these contingency tables, some ordinal dependent variables were recoded into dichotomous variables where “Always” and “Sometimes” were combined into one category, and “Never” was left as its own category. Any variables found to be significantly associated with the variables of interest were analysed through ordinal logistic regression, where the dependent variables were left in their original ordinal coding. It was ensured that the data met the assumptions of ordinal logistic regression through using Pearson’s chi-squared goodness-of-fit tests and tests of parallel lines. The level of significance was set at 0.05.

## Results

Of the 3013 ANC providers invited to participate, 408 responded. From those responses, seventeen were deleted due to incomplete (defined as less than 50% of the survey complete), duplicate and interstate surveys. This resulted in a total of 393 surveys (17.6% response rate) comprising of 124 GPs (15% response rate), 74 O&Gs (24.3% response rate) and 195 midwives (13.6% response rate). Missing data across the survey items ranged from 0.25 to 4.1%.

### Demographics

The respondents comprised of 85.1% females (*n* = 331) and 14.9% males (*n* = 58) with a mean age of 49.8 years. All ANC providers worked full time (>35 h/week) with most (88.2%) practising in the Sydney urban area. Respondents had an average of 20.2 years of experience in antenatal care with more than two-thirds (75.3%) having a postgraduate qualification. More than half (60.7%) reported encountering pregnant women with a dental problem every week while less than 30% provided dental referrals. Very few ANC providers (4.1%) reported receiving education/training on oral health care during pregnancy and only 16.7% had any brochures on oral health in their practice (Table 1).

**Table 1:** Characteristics of NSW ANC providers

	GPs No. (%)	O&Gs No. (%)	Midwives No. (%)	Total No. (%)
Age (Years) (Mean ± SD^a^)	50.7 ± 10.0	49.3 ± 12.5	49.5 ± 9.6	49.8 ± 10.3
Gender				
Male	29 (23.4%)	29 (40.3%)	0 (0%)	58 (14.9%)
Female	95 (76.6%)	43 (59.7%)	193 (100.0%)	331 (85.1%)
Work Sector				
Private Setting	113 (91.1%)	33 (45.8%)	14 (7.3%)	160 (41.1%)
Public Setting	11 (8.8%)	39 (54.2%)	179 (92.7%)	229 (58.9%)
Hours worked per week (Mean ± SD)	35.3 ± 14.2	47.8 ± 14.5	31.8 ± 10.3	36.0 ± 13.9
Location of practice				
Sydney & surrounding areas	106 (89.1%)	60 (81.1%)	156 (85.7%)	322 (88.2%)
Regional NSW	13 (10.9%	4 (5.4%)	26 (14.3%)	43 (11.8%)
Education – Highest Qualification				
Bachelors degree	32 (26.2%)	15 (20.8%)	47 (25.1%)	94 (24.7%)
Postgraduate diploma	57 (46.7%)	25 (34.7%)	81 (43.3%)	163 (42.8%)
Masters degree	23 (18.9%)	19 (26.4%	56 (29.9%)	98 (25.7%)
Doctorate degree	10 (8.2%)	13 (18.1%)	3 (1.6%)	26 (6.8%)
Years of experience (Mean ± SD)	22.1 ± 10.8	17.6 ± 12.7	19.9 ± 10.4	20.2 ± 11.1
Number of pregnant women encountered each week with oral health problems				
None	55 (45.8%)	25 (34.2%)	68 (37.0%)	148 (39.3%)
1–5	53 (44.2%)	39 (53.4%)	95 (51.6%)	187 (49.6%)
6–10	3 (2.5%)	3 (4.1%)	5 (2.7%)	11 (2.9%)
> 10	9 (7.5%)	6 (8.2%)	16 (8.7%)	31 (8.2%)
Number of dental referrals given to pregnant women each week				
None	80 (65.6%)	43 (58.9%)	149 (78.4%)	272 (70.6%)
1–5	40 (32.8%)	29 (39.7%)	38 (20.0%)	107 (27.8%)
6–10	1 (0.8%)	1 (1.4%)	0 (0%)	2 (0.5%)
> 10	1 (0.8%)	0 (0%)	3 (1.6%)	4 (1.0%)
Have you received formal education/training on ‘oral health care during pregnancy’				
Yes	2 (1.6%)	5 (6.8%)	9 (4.7%)	16 (4.1%)
No	120 (98.4%)	68 (93.2%)	184 (95.3%)	372 (95.9%)
Do you have any information/brochures on ‘oral health during pregnancy’ in your practice				
Yes	11 (9.0%)	6 (8.3%)	47 (24.7%)	64 (16.7%)
No	111 (91.0%)	66 (91.7%)	143 (75.3%)	320 (83.3%)

^a^SD: Standard deviation

### Main findings

#### Knowledge

The mean total number of correct responses among the 391 ANC providers for the 25 knowledge items was 16.6 (66.4%, SD 3.4). A high proportion of incorrect responses were recorded in relation to the impact of poor maternal oral health on pregnancy and infant outcomes (range 22.4–72.6% correct responses) and the appropriateness of certain dental procedures and radiographs during pregnancy (range 46.5–86.8% correct responses). The percentage of correct responses for each knowledge item is shown in Table 2.

**Table 2:** Percentage of correct responses to perinatal oral health knowledge items (*N* = 393) of ANC providers in NSW

Item Content (Correct answer)	Correct No. (%)
Pregnancy exacerbates existing dental problems: (True)	292 (74.5%)
Gingivitis is more serious than Periodontitis: (False)	199 (50.8%)
Calcium will be drawn out of mothers’ teeth by developing baby: (False)	164 (41.8%)
Gingivitis is a potentially reversible infection of the gums: (True)	354 (90.8%)
Poor maternal oral health can contribute to early childhood decay: (True)	242 (62.1%)
Periodontal disease has been associated with the following:	
• Stillbirth: (True)	155 (40.3%)
• Preterm delivery: (True)	283 (72.6%)
• Spontaneous abortion/miscarriage: (True)	195 (50.1%)
• Preeclampsia: (True)	87 (22.4%)
• Low birth weight: (True)	222 (57.2%)
Women should receive preventive dental care during pregnancy: (True)	361 (95.8%)
Basic dental treatment is safe during pregnancy: (True)	378 (96.9%)
It is unsafe to obtain dental radiographs in pregnant women: (False)	230 (59.7%)
Pregnant women should receive only emergency dental care: (False)	334 (86.1%)
Elective dental treatment should be delayed until after pregnancy: (True)	142 (36.8%)
These dental procedures are safe during pregnancy:	
• Extractions: (True)	294 (76.0%)
• Local anaesthetic: (True)	336 (86.8%)
• Root canal: (True)	181 (46.5%)
• Scaling and root planning: (True)	241 (62.1%)
These medications are safe during pregnancy:	
• Paracetamol: (True)	373 (94.9%)
• Aspirin: (False)	193 (49.9%)
• NSAIDs: (False)	269 (69.0%)
• Amoxicillin: (True)	366 (93.6%)
• Erythromycin: (True)	283 (73.1%)
• Doxycycline: (False)	258 (66.7%)

#### Attitude

The majority of ANC providers agreed that maintaining oral health during pregnancy was important (99%) and that pregnant women should receive a dental check early in their pregnancy (93.4%). However, less than a third felt they had the skills to provide oral health advice to pregnant women. In addition, 93.6% (*n* = 368) of respondents agreed that pregnant women are more likely to seek dental care if their ANC providers recommend it. Yet, over 50% stated that currently there is lack of understanding between ANC providers regarding dental care for pregnant women . Further, more than half of respondents (56.9%) agreed that the cost of dental treatment was a barrier to advising dental care during pregnancy. More than two-thirds were interested in further information and training on oral health care for pregnant women (Table 3).

**Table 3:** Attitudes to Perinatal Oral Health items of ANC Providers in NSW

Attitude	ANC *N* = 393 No. (%)		
	Agree	Not Sure	Disagree
Maintaining oral health during pregnancy is important	388 (99.0%)	2 (0.5%)	2 (0.5%)
Pregnant women should receive a dental check early in their pregnancy	367 (93.4%)	21 (5.3%)	5 (1.3%)
Pregnant women are more likely to seek dental care if their antenatal care providers recommend it	368 (93.6%)	18 (4.6%)	7 (1.8%)
Currently there is good understanding between ANC providers and dentists regarding dental care for pregnant women	51 (13.0%)	119 (30.3%)	223 (56.7)
There is insufficient time to advise pregnant women on oral health during antenatal visit	123 (31.6%)	42 (10.8%)	224 (57.6%)
Asking pregnant women about oral health is outside routine ANC practice	93 (23.8%)	32 (8.2%)	265 (67.9%)
Conducting a visual mouth examination during pregnancy is outside routine GP/O&G practice	217 (55.6%)	42 (10.8%)	131 (33.6%)
The cost of dental treatment is a barrier to advising pregnant women	222 (56.9%)	47 (12.1%)	121 (31.0%)
I have the skills to advise pregnant women on oral health	121 (31.0%)	107 (27.4%)	162 (41.5%)
Oral assessments of pregnant women at antenatal visits is important	234 (60.2%)	113 (29.0%)	42 (10.8%)
I feel confident about performing oral assessments	70 (17.9%)	77 (19.7%)	243 (62.3%)
Pregnant women will be comfortable with a GP/O&G assessing oral health during normal antenatal checkups	171 (43.8%)	137 (35.1%)	82 (21.0%)
The link betwen periodontal disease and preterm birth is too tenuous for me to warn pregnant women about it	32 (8.2%)	139 (35.6%)	219 (56.2%)
The link between dental caries in mothers and in babies is too tenuous for me to warn my patients about it	34 (8.7%)	142 (36.5%)	213 (54.8%)
I am concerned about being sued if something goes wrong in a pregnancy	92 (23.7%)	51 (13.1%)	245 (63.1%)
I am interested in further information about dental care to pregnant women	363 (92.8%)	13 (3.3%)	15 (3.8%)
I am interested in further training to provide dental assessments to pregnant women	297 (76.3%)	51 (13.1%)	41 (10.5%)
There is a need for universal guidelines for oral health care during pregnancy for all health professionals	367 (94.1%)	14 (3.6%)	9 (2.3%)

#### Practice

Only 16.4% (*n* = 62) of ANC providers stated that they always discuss the importance of oral health with pregnant women and 21.5% (*n* = 82) always advised women to visit dentists early in their pregnancy. Very few (<10%) respondents always provide counselling regarding the association between poor maternal oral health and birth outcomes/caries transmission and only 15% ask women specifically about their current oral health (Table 4).

**Table 4:** Frequency of Perinatal Oral Health Practices of ANC Providers in NSW

Practice	ANC *N* = 393 No. (%)		
	Always	Sometimes	Never
I discuss the importance of oral health with pregnant women during clinical care	62 (16.4%)	189 (49.9%)	128 (33.8%)
I advise pregnant women to delay dental visits until after pregnancy	8 (2.1%)	41 (10.8%)	332 (87.1%)
I advise pregnant women to visit dentists during early pregnancy	82 (21.5%)	183 (48.0%)	116 (30.4%)
I provide counselling regarding the association of poor periodontal health with negative birth outcomes	32 (8.4%)	142 (37.3%)	207 (54.3%)
I provide counselling regarding caries prevention and transmission	28 (7.3%)	134 (35.2%)	219 (57.5%)
I ask pregnant women about current oral health	56 (14.9%)	155 (41.1%)	166 (44.0%)
I ask specific questions related to oral health practices	29 (7.6%)	128 (33.6%)	224 (58.8%)
I conduct visual examination (of the mouth) of pregnant women as part of antenatal care	40 (10.6%)	83 (21.9%)	256 (67.5%)

#### Barriers

The main barriers for ANC providers in this area were the lack of practice guidelines on oral health care during pregnancy in Australia (81%), insufficient training to perform oral health assessments on pregnant women (80.6%) and the high cost of dental treatment for pregnant women (71.9%). Other relevant barriers included lack of time to provide oral health advice to pregnant women (59.0%), concerns about the safety of dental procedures during pregnancy (63.2%) and lack of knowledge about the risks involved when providing dental treatment during pregnancy (53.9%) (Table 5).

**Table 5:** Frequency of responses to Perinatal Oral Health Care Barriers for ANC providers from NSW

Barriers	ANC *N* = 393 (%)		
	Agree	Not Sure	Disagree
Lack of time providing advice about oral health care to pregnant women	227 (59.0%)	51 (13.2%)	107 (27.8%)
Asking about oral health questions is of a sensitive nature	72 (18.7%)	30 (7.8%)	284 (73.6%)
Inability for pregnant women to pay for dental care	277 (71.9%)	51 (13.2%)	57 (14.8%)
Concern of pregnant women about safety of dental procedures	242 (63.2%)	60 (15.7%)	81 (21.1%)
My lack of knowledge of risks involved when treating pregnant women	207 (53.9%)	6617.2%)	111 (28.9%)
Lack of knowledge of importance of oral health during pregnancy	185 (48.1%)	44 (11.4%)	156 (40.5%)
Lack of practice guidelines on oral health care during pregnancy in Australia	312 (81.0%)	44 (11.4%)	29 (7.5%)
Not sufficiently trained to perform oral health assessments on pregnant women	311 (80.6%)	43 (11.1%)	32 (8.3%)
Peer pressure from colleagues	21 (5.5%)	104 (27.0%)	260 (67.5%)
Patient lack of concern with oral health care during pregnancy	205 (53.5%)	80 (20.9%)	98 (25.6%)
Reluctance among dental professinals to treat pregnant women	105 (27.3%)	198 (51.6%)	81 (21.1%)
Risk of labour in dental practice	27 (7.0%)	123 (32.0%)	234 (60.9%)
Legal risks associated with negative birth outcomes	50 (13.0%)	156 (40.6%)	178 (46.4%)

### Factors predicting perinatal oral health practice of ANC providers in NSW

Significant positive correlations were found between the practice variable “I discuss the importance of oral health with pregnant women” and average years of experience (*p* = 0.001), receiving formal education on oral health care during pregnancy (*p* = 0.001), having information brochures on oral health care during pregnancy (*p* = <0.0001) and all items from the knowledge section of the survey (*p* = 0.003–0.049). Positive correlations were also evident between the variable “I advise pregnant women to visit dentists during early pregnancy” and average years of experience (*p* = 0.0002), having information brochures on oral health care during pregnancy (*p* = <.0001) and all knowledge items (*p* = .0003–0.20). There was also significant correlation between ANC providers self-reported knowledge and routine referral of pregnant patients to a dentist (*p* = 0.025).

Further analysis using ordinal logistic regression modeling showed that ANC providers were more likely to discuss the importance of oral health with pregnant women if they had received formal education/training on oral health during pregnancy (OR 3.51), received information/brochures on oral health (OR 3.04) or had more than 20 years of experience (OR 2.61). ANC providers were also more likley to advise pregnant women to visit dentists during early pregnancy if they had information/brochures on oral health during pregnancy (OR 4.53), a knowledge item score of greater than 75% (OR 2.38), or greater than 20 years of experience (OR 3.55) (Table 6).

**Table 6:** Predictors of ANC providers discussing the importance of oral health with pregnant women and advising women to visit dentists during early pregnancy

Predictors of ANC providers discussing importance of oral health with pregnant women		Predictors of ANC providers advising women to visit dentists during early pregnancy	
Years of experience	Odds Ratio (95% CI)	Years of experience	Odds Ratio (95% CI)
<=10 yrs vs >20 yrs	2.617 (1.600, 4.280)^†^	<=10 yrs vs >20 yrs	3.554 (2.160, 5.842) †
<=10 yrs vs 11–20 yrs	1.259 (0.719, 2.197)	<=10 yrs vs 11–20 yrs	2.098 (1.201, 3.666) ^†^
Received formal education / training on oral health during pregnancy	3.518 (1.246, 9.924) ^†^	Has information / brochures on oral health during pregnancy	4.536 (2.593, 7.933) ^†^
Has information / brochures on oral health during pregnancy	3.047 (1.740, 5.333) ^†^	Knowledge < 75% correct responses	2.387 (1.543, 3.691) ^†^
Nagelkerke R-squared	0.13	Nagelkerke R-squared	0.194
Pearson goodness-of-fit	16df (*p* = 0.693)	Pearson goodness-of-fit	18df (*p* = 0.789)
Test of parallel lines	4df (*p* = 0.900)	Test of parallel lines	4df (*p* = 0.745)

^†^ *P* < 0.05

## Discussion

This cross-sectional survey aimed to explore the current knowledge, attitudes, practices and barriers for ANC providers in NSW regarding oral health care during pregnancy. We also sought to identify predictors of likelihood of ANC providers discussing oral health and providing referrals to pregnant women. This is the first time such a study has been conducted in Australia, with the only previous study being a qualitative study exploring the perceptions of midwives in NSW regarding maternal oral health [25].

### NSW sample

The mean age of the study sample was fairly comparable with population data for GPs (50.7 vs 50.5) [28] and O&Gs (49.3 vs 52.1) [29] but was slightly higher for midwives (49.5 vs 45.7). Similarly, the location of the antenatal practice (Sydney and surrounding vs. Regional NSW) showed similar trends to population data for GPs (88.2 vs 11.8% compared to 93.6 vs 6.4%) [30] and O&Gs (81.1 vs. 5.4% compared to 88.7 vs. 11.3%) [31].

### Perinatal oral health knowledge of ANC providers

Overall all ANC providers displayed adequate knowledge of the importance and safety of oral care in pregnancy. Similar findings were reported among O&Gs in the USA, with the majority recognising the importance of receiving dental care during pregnancy [32]. Interestingly, the previous qualitative Australian study [25] reported that midwives were largely unaware of the importance of maintaining oral health during pregnancy. Further exploration of the data showed that the underlying knowledge of the importance of oral health care in pregnancy was tenuous, with 22–57% of ANC providers not having a firm understanding of the impact of periodontitis on birth outcomes other than preterm delivery. These results were similar to those from an earlier study [32] in the United States which also reported poor knowledge of the association of periodontal disease with pregnancy outcomes, with only some (9–42%) O&Gs identifying negative birth outcomes. Other studies involving GPs [33–39] also support these findings, reporting that 40–70% of GPs were unaware of associations between oral health and pregnancy outcomes. Additionally in our study, some ANC providers reported limited knowledge regarding the association between maternal oral health and early childhood caries in their infants, with less than two thirds of providers identifying this. Earlier studies have reported even lower knowledge of this association among O&Gs with less than a third of respondents identifying this link [32, 34].

Our findings also support earlier reports [32–34, 40] about the misconceptions among ANC providers regarding oral health in pregnancy. Over half of ANC providers believed the myth that calcium will be drawn out of mothers’ teeth by the developing baby, and over a third falsely believed it is unsafe to obtain dental radiographs in pregnant women. The respondents did however demonstrate sound knowledge about the safety of dental procedures during pregnancy, similar to previous studies [32, 40, 41]. Knowledge of safe medications during pregnancy was poor among ANC providers. Between 30-50% of ANC providers incorrectly identified medications such as Aspirin, NSAIDs and Doxycycline as safe during pregnancy, with doxycycline incorrectly being identified as safe by one third of providers. In contrast, a study in the United States (32) reported only 1% of respondents incorrectly identifying tetracyclines as safe during pregnancy. A possible reason for this discrepancy could be that respondents to our surveys did not recognise doxycycline as a tetracycline antibiotic. Nevertheless, this incorrect understanding has serious implications, as the administration of doxycycline can have adverse effects on the growing foetus [42]. There may also have been confusion over the use of Aspirin as well due to fact it is now recommended routinely for some pregnant women such as those with a prior history of pre-eclampsia.

Areas of poor knowledge in this study are of concern as knowledge was significantly associated with the provision of dental referrals, suggesting good dental knowledge is a facilitator for the appropriate referral of pregnant women to dental services. This is supported by findings from a study in United States [43] which show a significant correlation between knowledge and provision of dental services. The poor knowledge among NSW ANC providers identified in our study could be due to a lack of emphasis has placed on oral health care during pregnancy in Australia [19]. It was not until 2011 that National Antenatal Care Guidelines in Australia advocated the need for oral health care during pregnancy [44]. Furthermore, apart from an oral health professional training program for midwives [22] there appears to be little training available for other ANC providers in Australia nor is training in oral health care a strong feature of undergraduate medicine and midwifery programs [20]. The United States has already been addressing this issue since 2010 by combining some of the coursework of medical and dental students, allowing them to collaborate and learn about the importance and role of each discipline in patient care [45–47]. Although efforts are currently underway in Australia to educate midwifery students in this area (Duff M, Dahlen, M., Burns, E., Priddis, H., Schmeid, V.: George A Designing an Oral Health Module for the Bachelor of Midwifery Program at an Australian University, under review) more emphasis on oral health care in pregnancy is required at an undergraduate level across other disciplines.

### Attitude of ANC providers towards perinatal oral health

In this study, ANC providers displayed positive attitudes towards oral health during pregnancy with nearly all acknowledging that maternal oral health care is important as well as the significance of their role in this area. Similar findings were reflected in the earlier exploratory work [25] and internationally [41, 48]. However like Strafford and colleagues in their study involving obstetricians [41] our study shows that these positive attitudes are not being translated into practice among ANC providers. Between 30 and 70% of participants never engaged in practices to address the oral health of pregnant women, which confirms earlier reports that oral health is not being widely addressed during antenatal care [19, 25, 32, 33]. In addition, a number of participants agreed that there was divergent agreement between ANC providers and dentists regarding perinatal oral health care. This view could stem from the fact that dentists, due to various reasons, are sometimes hesitant to treat pregnant women [20, 24].

### Predictors and barriers of perinatal oral health practices among ANC providers

Our findings indicate that ANC providers who are more knowledgeable about maternal oral health, have training and information in this area and have greater experience are more likely to engage in practices addressing the oral health of pregnant women. Further, the key barriers cited by participants in this area were a lack of practice guidelines on oral health care during pregnancy (81%) and not being sufficiently trained to perform oral health assessments on pregnant women (80%). The majority of ANC providers were interested in further information about maternal oral health and training to provide dental assessments to pregnant women.

Overall this study provides evidence that ANC providers in NSW are not emphasising oral health in their practice. Although most agree that pregnant women should receive a dental check early in their pregnancy and acknowledge their important roles in this area, they appear to lack the skills and knowledge to provide oral health advice to pregnant women. There also appears to be a lack of consensus regarding perinatal oral health care among ANC providers and dentists in NSW. Further education and training in perinatal oral health is needed for ANC providers along with greater dissemination of oral health promotional information. Development of comprehensive perinatal oral health guidelines similar to other countries [16] may improve consensus among health professionals and increase the likelihood that ANC providers engage in positive practices to promote oral health among pregnant women.

### Limitations

This survey sought to obtain responses from three groups of ANC providers. The response rates for each of these groups varied from 13.6 to 24.3%. These rates are low, and therefore the findings may not reflect those of all ANC providers, or indeed specific groups of ANC providers, within New South Wales. Nonetheless, the demographic characteristics of the sample of GPs and O&Gs were similar to the NSW population, while the midwives were slightly older. Contemporary authors highlight this global problem [49] which is reflected in time-poor clinicians who have difficulty completing online or hardcopy surveys. Strategies to improve the response rates will be explored in the subsequent national survey of ANC providers which is forthcoming.

## Conclusion

In summary, from these preliminary findings from ANC providers in NSW, it appears that there is willingness among ANC providers, if appropriately educated and trained in oral health assessment, to encourage pregnant women to focus on their oral health and where appropriate seek dental health services. Guidelines, multimedia tools and available brochures and other education strategies could mobilize ANC providers throughout NSW to support perinatal oral health. Misconceptions are evident although limited in number. Barriers such as the cost of access to dental health services are more difficult to address and would require state or national funding initiatives for those women unable to pay for these expensive services or unable to access public dental services. Further research at a national level is required to identify whether these findings are similar in all Australian states and territories. Nonetheless the development of consensus guidelines relating to perinatal oral health in Australia requires urgent attention and active lobbying by key professional bodies. A process of guideline development that brings together all ANC providers as well as private and publicly funded dental services is likely to engage all ANC providers throughout Australia in this important health strategy for Australian women and their infants.

## Abbreviations

ANC: Antenatal care providers
GPs: General practitioners
O&Gs: Obstetricians and gynaecologists
NSW: New South Wales
